# Which missing value imputation method to use in expression profiles: a comparative study and two selection schemes

**DOI:** 10.1186/1471-2105-9-12

**Published:** 2008-01-10

**Authors:** Guy N Brock, John R Shaffer, Richard E Blakesley, Meredith J Lotz, George C Tseng

**Affiliations:** 1 Department of Bioinformatics and Biostatistics, School of Public Health and Information Sciences, Universtiy of Louisville, Louisville, KY 40292, USA; 2 Department of Human Genetics, University of Pittsburgh Graduate School of Public Health, Pittsburgh, PA 15261, USA; 3 Department of Biostatistics, University of Pittsburgh Graduate School of Public Health, Pittsburgh, PA 15261, USA; 4 Department of Computational Biology, University of Pittsburgh School of Medicine, Pittsburgh, PA 15213, USA

## Abstract

**Background:**

Gene expression data frequently contain missing values, however, most down-stream analyses for microarray experiments require complete data. In the literature many methods have been proposed to estimate missing values via information of the correlation patterns within the gene expression matrix. Each method has its own advantages, but the specific conditions for which each method is preferred remains largely unclear. In this report we describe an extensive evaluation of eight current imputation methods on multiple types of microarray experiments, including time series, multiple exposures, and multiple exposures × time series data. We then introduce two complementary selection schemes for determining the most appropriate imputation method for any given data set.

**Results:**

We found that the optimal imputation algorithms (LSA, LLS, and BPCA) are all highly competitive with each other, and that no method is uniformly superior in all the data sets we examined. The success of each method can also depend on the underlying "complexity" of the expression data, where we take complexity to indicate the difficulty in mapping the gene expression matrix to a lower-dimensional subspace. We developed an entropy measure to quantify the complexity of expression matrixes and found that, by incorporating this information, the entropy-based selection (EBS) scheme is useful for selecting an appropriate imputation algorithm. We further propose a simulation-based self-training selection (STS) scheme. This technique has been used previously for microarray data imputation, but for different purposes. The scheme selects the optimal or near-optimal method with high accuracy but at an increased computational cost.

**Conclusion:**

Our findings provide insight into the problem of which imputation method is optimal for a given data set. Three top-performing methods (LSA, LLS and BPCA) are competitive with each other. Global-based imputation methods (PLS, SVD, BPCA) performed better on mcroarray data with lower complexity, while neighbour-based methods (KNN, OLS, LSA, LLS) performed better in data with higher complexity. We also found that the EBS and STS schemes serve as complementary and effective tools for selecting the optimal imputation algorithm.

## Background

As with many types of experimental data, expression data obtained from microarray experiments are frequently peppered with missing values (MVs) that may occur for a variety of reasons. Randomly scattered MVs may be due to spotting problems, poor hybridization, inadequate resolution, fabrication errors, or contaminants on the chip including scratches, dust, and fingerprints. Because many down-stream microarray analyses such as classification methods, clustering methods, and dimension reduction procedures require complete data, researchers must either remove genes with one or more MVs, or, preferably, estimate the MVs before such procedures can be employed. Consequently, many algorithms have been developed to accurately impute MVs in microarray experiments [1-6].

The first evaluation of MV estimation methodology in microarray data was reported by Troyanskaya et al. [1], who compared a variety of algorithms and concluded that two methods, k-Nearest-Neighbors (KNN) and singular value decomposition (SVD), performed well in their test data sets. Others have developed more sophisticated algorithms and shown that in some situations, these variants outperform KNN [7-12]. Although one study [4] evaluated the performance of their method along with a few others over seven microarray data sets, typically these reports have employed a limited number of data sets to evaluate their methods. Another study has assessed the performance of imputation methods on a pair of data sets with strong and weak correlation structure, respectively, and concluded that the preferred choice of method and parameters are different for each set of data and dependent on the structure of expression matrix [13].

In this study, we present a comprehensive evaluation of the performance of current imputation methods across a wide variety of types and sizes of microarray data sets, to assess their performance under different conditions and establish guidelines for their appropriate use. In addition, we develop and test two selection procedures for determining the most appropriate imputation method for a given data set. To this end, we have implemented and tested existing methods for MV imputation, to assess the performance of each of these methods under various conditions and determine the circumstances for which different imputation procedures are preferred.

Specifically, we tested eight different algorithms from the literature that have been shown to perform well at imputing MVs in microarray data sets: KNN.e (Euclidean based neighbor selection), KNN.c (correlation based neighbor selection), SVD, ordinary least squares (OLS) [8,9], partial least squares (PLS) [8], Bayesian principal component analysis (BPCA) [2], local least squares (LLS) [10], and least squares adaptive (LSA) [9]. We compared the performance of these methods on nine data sets of various sizes, for different percentages of missing data, and under varying algorithm parameters. Based on this evaluation we proposed two selection procedures, entropy-based selection (EBS) and self-training selection (STS), for determining the most appropriate method for new data. EBS determines the optimal method via an entropy measure of data "complexity", and a linear model is fitted using the nine selected data sets for prediction. The complexity of a data set is a measure of the difficulty in mapping the data set to a lower-dimensional subspace. Computation of this procedure is fast once the model is fitted, but also more dependent on the selection of data sets in the model fitting. STS, on the other hand, performs self-training simulation. Its computation is more intensive but the performance is better. The STS scheme outperforms any single imputation method, and combining the two complementary schemes presents an appealing solution for MV imputation of microarray data.

## Results

### Optimization of parameters of each method

Optimal parameter values for the eight methods under investigation, for each data set, are reported in Table 1. These optimal values were determined using a set of initial simulations (Simulation I – see Methods for description). For the nieghbor-based imputation methods (KNN.e, KNN.c, OLS), the optimal number of neighbors was generally in the range of 10 to 20 and is consistent with previous investigations [1,8,9]. The range of components selected for PLS was between 8 and 15, while SVD typically used a percentage of eigenvalues between 0.15 and 0.25. LLS has its parameter optimization built into the algorithm, but we report the optimal values determined by the method, which we held fixed for the remaining simulations. Although the optimal *k *values varied greatly (between 210 and 2397), the number of tested *k *values in this range was typically around 6 or 7. Neither the LSA nor BPCA methods required parameter optimization, as the parameter settings are predetermined in both cases.

**Table 1:** Optimum parameter values for the imputation methods in our study. BPCA and LLS did not require parameter optimization.

Method	Parameter/Values tested	Optimal Value								
		ALI	ALO	BAL	CAU	GAS	GOL	ROS	SP.AFA	SP.ELU
KNN.e	K = (5,10,15,25,50,100,200)	5	5	10	15	10	15	5	10	15
KNN.c	K = (5,10,15,25,50,100,200)	10	10	15	15	10	15	5	15	25
OLS.c	K = (5,10,15,25,50,100,200)	10	10	10	15	5	10	5	25	200
PLS	C = (2,3,4,5,6,7,8,9,10,15,25)	8	10	15	8	10	5	15	8	9
SVD	p = 0.05 to 0.5, by 0.05	0.1	0.25	0.2	0.25	0.15	0.15	0.15	0.2	0.15
LLS	k (values tested depended on data set)	710	710	710	2397	2397	1598	710	210	210

### Performance of the imputation methods

**Figure 1:**
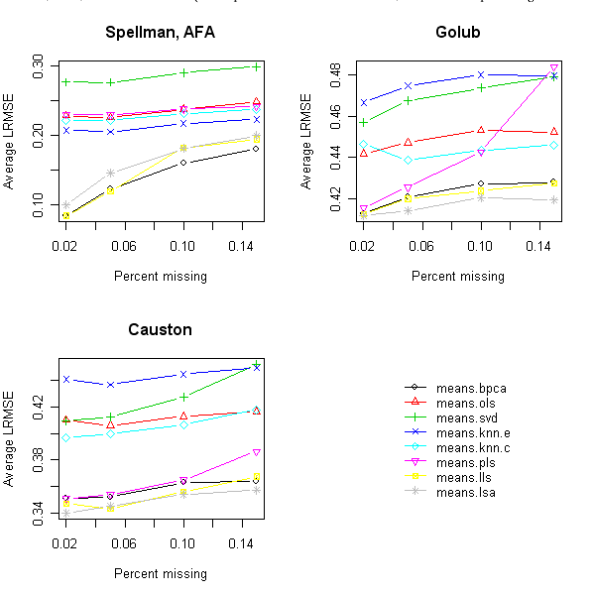
Average LRMSE values for different percentages of missing values in different microarray data sets.

Figure 1 plots the performance of each method as a function of the percentage of MVs (2%, 5%, 10%, and 15%) for the SP.AFA, GOL, and CAU data sets (one representative each from the TS, ME, and ME × TS categories, respectively). The performance is judged by the log-transformed root mean squared error (LRMSE), and tends to decrease with increasing percentage of MVs for each method. The relative performance of the imputation methods did not vary much with the percentage of MVs, although the performance of PLS did deteriorate drastically with a higher percentage of MVs in the GOL data. Also, to a lesser extent, the performance of LLS dropped off with higher percentage of MVs. Although this drop-off was slight, it did influence the method selection in the STS scheme. Bø *et al.*[9] have previously reported that in microarray experiments, the majority of genes contain less than 5% MVs. Hence, in our simulations, we fixed the percentage of MVs at 5% when evaluating the imputation methods and selection schemes.

**Figure 2:**
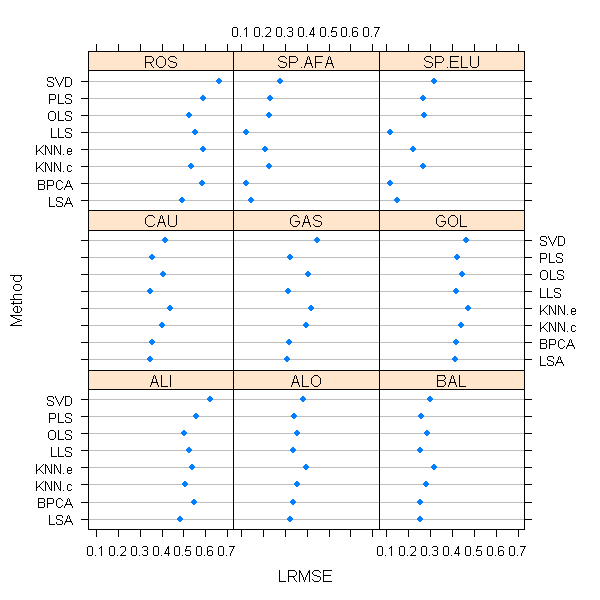
Average LRMSE values for all imputation methods and all data sets, using the optimized parameter values and with 5% missing.

The average LRMSE values for each method in all nine data sets are given in Figure 2. There is significant variation in imputation success (LRMSE values) across data sets and imputation methods, although the 3 top performing methods (LSA, LLS, and BPCA) were all very competitive with each other. The overall ranking for each method on each data set is given in Table 2. Overall, LSA performed the best, followed by LLS and then BPCA. The differences between these methods, though statistically significant (p < 0.01 after adjustment for multiple comparisons), were slim (difference of 0.007 LRMSE value for LSA vs. LLS and 0.008 for LLS vs. BPCA), especially compared to the differences between these methods and the remaining methods. Further, it should be noted that no single method uniformly outperformed the others, so that a unanimous "best method" cannot be declared.

**Table 2:** Average LRMSE values, ranking (in parenthesis), and regression slope ($\hat{\beta }$) in equation (5), for the eight methods under investigation (from Simulation II). An asterisk indicates a *p*-value less than 0.001.

Method	ALI	ALO	BAL	CAU	GAS	GOL	ROS	SP.AFA	SP.ELU	Overall	Slope
LSA	0.487 (1)	0.323 (1)	0.253 (1)	0.348 (2)	0.311 (1)	0.414 (1)	0.492 (1)	0.147 (3)	0.150 (3)	0.325	-0.292*
LLS	0.550 (4)	0.339 (2)	0.254 (2)	0.355 (1)	0.316 (2)	0.421 (2)	0.584 (4)	0.122 (1)	0.117 (1)	0.332	-0.125*
BPCA	0.524 (6)	0.338 (3)	0.254 (3)	0.346 (3)	0.315 (3)	0.420 (3)	0.555 (5)	0.120 (2)	0.115 (2)	0.340	0.007
PLS	0.538 (7)	0.397 (4)	0.316 (4)	0.439 (4)	0.418 (4)	0.473 (4)	0.591 (6)	0.208 (7)	0.220 (6)	0.373	0.330*
KNN.c	0.509 (3)	0.354 (5)	0.280 (5)	0.402 (5)	0.398 (5)	0.439 (5)	0.535 (3)	0.225 (5)	0.267 (5)	0.379	-0.082*
OLS	0.558 (2)	0.343 (6)	0.259 (6)	0.357 (6)	0.322 (6)	0.425 (6)	0.590 (2)	0.232 (6)	0.268 (7)	0.382	-0.149*
KNN.e	0.503 (5)	0.356 (8)	0.286 (8)	0.408 (8)	0.406 (7)	0.448 (8)	0.527 (7)	0.229 (4)	0.273 (4)	0.400	-0.237*
SVD	0.622 (8)	0.385 (7)	0.301 (7)	0.415 (7)	0.446 (8)	0.465 (7)	0.661 (8)	0.279 (8)	0.316 (8)	0.432	0.547*

**Figure 3:**
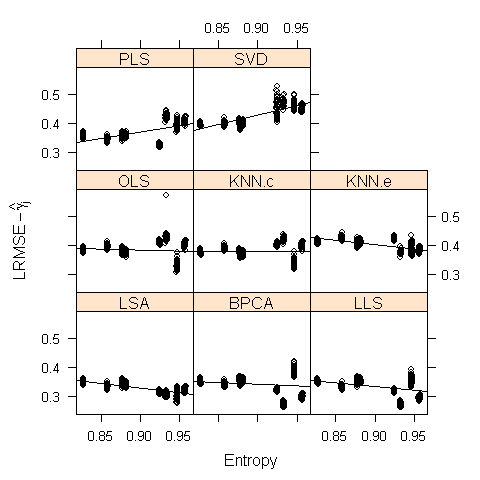
Plot of entropy vs. adjusted LRMSE values ($\text{LRMSE}-{\hat{\gamma }}_{J}$) for each imputation method and each data set using Simulation II, with fitted regression lines.

The entropy of each data set, calculated using (4), is given in Table 3. Contrary to what was expected, there was no observable correlation between the entropy level and experiment type (TS, ME, and TS × ME). Figure 3 shows the relationship between the entropy of each data set and the performance ($\text{LRMSE}-{\hat{\gamma }}_{J}$) for the imputation methods from Simulation II. The performance of many of the imputation methods was highly dependent on the entropy of the data, as indicated by the significance of the regression slope ${\hat{\beta }}_{M_{i}}$ associated with each method (see Table 2). For example, KNN.e performed well in high entropy data and poorly in low entropy data ($\hat{\beta }$ << 0), whereas PLS and SVD performed well for low but not high entropy data ($\hat{\beta }$ >> 0). The result of SVD is expected, since SVD essentially relies on successful dimension reduction, which corresponds exactly with the definition of the entropy measure. BPCA also relies on dimension reduction, but the probabilistic model shrinks the principal axes that are not relevant for imputation. Therefore, BPCA is quite robust to changes in the data complexity, and its performance is relatively stable over the range of entropy values. However, in the two highest entropy data sets (ALI and ROS), LSA and most of the other local-imputation methods outperform BPCA, the best global-method, by a relatively wide margin. Hence when the entropy measure of the data is high, local methods appear better suited to imputation than the global methods.

**Table 3:** Accuracy of the EBS method (Simulation II) and STS method (Simulation III). For Simulation II, the top performing imputation method and the predicted method by EBS during cross-validation is given for all 50 simulations. For Simulation III the optimal method, along with the STS selected method, is given for the 10 "first tier" simulations. In each case the number of simulations each method achieved the given ranking is given in parenthesis.

		Simulation II			Simulation III		
Data set	Entropy	Optimal	EBS	Accuracy	Optimal	STS	Accuracy
BAL	0.819	LSA (38), LLS (12)	LSA (50)	76%	LSA (9), LLS (1)	LSA (10)	90%
CAU	0.838	LLS (45), LSA (5)	LSA (50)	10%	LLS (10)	LSA (10)	0%
ALO	0.872	LSA (50)	LSA (50)	100%	LSA (10)	LSA (10)	100%
GOL	0.876	LSA (50)	LSA (50)	100%	LSA (10)	LSA (10)	100%
SP.ELU	0.909	LLS (41), BPCA (9)	LSA (50)	0%	LLS (10)	BPCA (10)	0%
GAS	0.911	LSA (50)	LSA (50)	100%	LSA (10)	LSA (10)	100%
SP.AFA	0.94	LLS (40), BPCA (10)	LSA (50)	0%	LLS (9), BPCA (1)	BPCA (10)	10%
ROS	0.944	LSA (50)	LSA (50)	100%	LSA (10)	LSA (10)	100%
ALI	0.947	LSA (50)	LSA (50)	100%	LSA (10)	LSA (10)	100%
					
			Overall	65%		Overall	67%

### Performance of EBS

As indicated in Figure 3 and Table 2, the performance of the imputation methods varied with the complexity of the data. The EBS scheme makes use of this relationship to select the best imputation method for a given data set. The accuracy of the EBS scheme in selecting the top performing method as determined by the leave-one-out cross validation using Simulation II is given in Table 3. Since the LSA algorithm was the top performer in the majority of the simulations, and the second or third best in the remainder, it is not surprising that the EBS scheme ended up selecting this method for every data set. In cases where the LSA algorithm was not optimal, the LLS algorithm was either the top or second best performer, and the EBS scheme selected this algorithm as second best in those cases. Thus, one of the top two selected methods by EBS was always among the first or second best performing algorithms. Overall the linear model and EBS scheme provide deep insight into the MV imputation problem. This information alone, however, is not quite enough to support an effective selection of an MV imputation method.

### Performance of STS

The accuracy of the STS scheme in selecting the top performing method is given in Table 3. Though the overall accuracy of the STS scheme was only slightly higher than the EBS scheme, in cases where the two schemes differed (SP.AFA and SP.ELU), the STS scheme selected methods that were closer to the optimal imputation method. This is reflected in Table 4, which compares the average LRMSE scores based on the STS scheme with the gold standard optimal method and the EBS method. The STS scheme was significantly closer to the optimal LRMSE score than the EBS scheme (average difference of 0.001 vs. 0.0074, respectively), although the STS scheme was still significantly different from the optimal LRMSE value (95% CI for mean difference of (0.0007, 0.0014), p < 0.001, paired t-test). However, the STS scheme CI for the mean difference (between the LRMSE of the selected method and the optimal LRMSE) is an order of magnitude closer to zero compared to the EBS scheme CI. Using ten "second-tier" simulations was sufficient to determine the best imputation method, as in every case the Friedman test of equality between the rank-sum statistics of the methods was decidedly rejected, with *p*-values on the order of 10^-10 ^to 10^-12^.

**Table 4:** Average LRMSE values for each data set using the gold standard optimal imputation method, the STS selected method, and the EBS selected method for the "first tier" simulations in Simulation III. The difference in LRMSE values between the two selection schemes and the optimal method are also indicated, with an asterisk indicating a p-value below 0.05.

	Simulation III		
Data Set	Optimal (OPT)	STS (Difference)	EBS (Difference)
ALI	0.487	0.487 (0.0)	0.487 (0.0)
ALO	0.323	0.323 (0.0)	0.323 (0.0)
BAL	0.251	0.251 (6.04e-05)	0.251 (6.04e-05)
CAU	0.345	0.348 (2.76e-03*)	0.348 (2.76e-03*)
GAS	0.310	0.310 (0.0)	0.310 (0.0)
GOL	0.414	0.414 (0.0)	0.414 (0.0)
ROS	0.495	0.495 (0.0)	0.495 (0.0)
SP.AFA	0.117	0.121 (3.75e-03*)	0.145 (0.0284*)
SP.ELU	0.116	0.118 (2.73e-03*)	0.151 (0.0357*)

Overall Mean Difference	STS vs OPT:	0.0010* (0.0007, 0.0014)	
	EBS vs OPT:	0.0074* (0.0046, 0.0102)	
	STS vs EBS:	0.0064* (0.0038, 0.0090)	

## Discussions and Conclusion

We performed an extensive evaluation of existing methods for imputing missing values in microarray data. In contrast to the recent comprehensive comparative study in gene clustering of microarray data [14], where the order of performance of the methods was consistent across all investigated simulated and real data sets, our investigation demonstrates that the optimal imputation algorithms are all highly competitive with each other, and that no method is uniformly superior. The imputation method most commonly employed by researchers, KNN, was clearly bested by the more sophisticated algorithms we tested (LSA, LLS, BPCA, PLS). Therefore, KNN should not be the default choice for imputing MVs. Moreover, Troyanskaya *et al.*[1] found that neighbor selection based on the Euclidean distance was favorable, whereas we found that correlation based neighbor selection outperformed Euclidean neighbor selection in all but two cases (SP.ELU and SP.AFA). Thus, correlation based neighbor selection appears to be more robust to varying levels of complexity in the data.

Overall, the LSA, LLS, and BPCA imputation algorithms performed the best in our simulation study. Both the LSA and LLS algorithms are based on selection of gene neighbors for imputation, but they also each have features which resemble global based imputation. LSA uses array-based imputation in addition to gene-based imputation, and LLS allows for the selection of a very large number of genes (upwards of several thousand) to use for imputation. Since LLS solves the least squares system using the Moore-Penrose pseudoinverse of the neighbor gene expression matrix, singular matrices can be used. The pseudoinverse is determined by the SVD of the expression matrix, and this method is similar to the supervised principal components procedure [15], which has been shown to be successful in other genomic applications. LSA is more consistent than the other algorithms over the data sets we investigated (worst performance of 3^rd ^best in SP.AFA and SP.ELU), and thus has the overall advantage. In particular, the difference between LSA and LLS occurs mainly on the high entropy data sets ROS and ALI, where the performance of LLS drops considerably (to 4^th ^best out of 8), while LSA has the lowest LRMSE. However, we again emphasize that the overall differences between these top three (LSA, LLS, and BPCA) methods is slim, and that there is no conclusively best method. Several extensions and improvements from these methods have been proposed in the literature [3,4,11,12] and may outperform the methods we evaluated in this study.

For each imputation method, we used the optimal parameter settings for each data set. Generally, the parameter settings were similar across all data sets. For KNN and OLS, the number of neighbors selected was between 5 and 15, although in one case 200 neighbors was the optimal choice. For PLS, the number of components was between 8 and 15, and for SVD the percentage of eigenvalues was typically in the range of 0.15 to 0.25. The variation in the optimal parameter setting was greatest for LLS, though this range actually corresponded to only a small number of actual choices, and further the LRMSE curve was relatively flat over that range of values. It should be noted that neither BPCA nor LSA required parameter optimization, and LLS has parameter selection built into the algorithm internally, making these algorithms attractive choices for automated imputation of MVs.

While we have evaluated a broad spectrum of imputation algorithms in the literature, our coverage is by no means exhaustive. Some methods we excluded include [3], which uses non-negative least squares, projection onto complex sets (POCS) [4], which can include biological knowledge in a set theoretic framework, and [5], which uses gene ontology information in the MV imputation. In particular, Gan *et al.*[4] compare POCS with LSA on seven different data sets, and find that POCS performs as well or better than LSA in all cases. However, POCS has many parameters that the user must set or optimize over, making replication of the algorithm's success difficult. The algorithms we selected have repeatedly been shown to be successful in multiple studies, and are also easy to use and available from the web. Further, the EBS and STS selection schemes can in principle be used with any imputation algorithms.

Our assessment of the imputation algorithms and selection schemes is based on an overall average of the LRMSE. Other authors have examined the distribution of squared imputation errors (imputed value – true value) as a function of the true gene expression values to determine if certain algorithms are more accurate for imputing high (or low) expression levels [8]. For example, the results of Nguyen *et al.*[8] indicate that KNN does not perform well in the tails of the MV distribution. Others have assessed the impact of MV imputation on downstream analysis such as detection of differentially expressed genes and cluster analysis [11,16,17]. Though it is possible to incorporate this type of information into the selection schemes (for example, by using quantile regression to incorporate the distribution of imputation errors in the case of the EBS scheme), future study is needed to determine if this information would prove useful in selection of MV imputation algorithms.

Previous studies have shown that the performance of an imputation algorithm depends on the underlying correlation structure of the gene expression matrix [13]. We have further developed this idea by proposing an entropy measure which succinctly captures the complexity of the expression matrix. This measure (4) summarizes the correlation structure of the data via the dispersion of the eigenvalues of the covariance matrix. Low entropy indicates that the gene expression values are strongly correlated and that the data can be reduced to a lower dimensional space. In contrast, high entropy indicates complex data with local substructure, which cannot effectively be reduced to a lower dimensional space. The global-based imputation methods (PLS, SVD) performed better on microarray data with lower complexity, as evidenced by the positive regression coefficient associated with each method in the regression model (5). In contrast, neighbour-based methods (KNN, OLS, LSA, LLS) performed relatively better in high entropy data, and have negative regression coefficients in the model (5). These findings correspond with those found by [11]. The top three performing algorithms (LSA, LLS, and BPCA) are all highly competitive with each other, and are less sensitive to changes in data complexity than the remaining algorithms. However, the gap between LSA and the global imputation algorithms increases for the highest entropy data sets (ALI and ROS). One notable exception to the success of LSA are the SP.AFA and SP.ELU data sets, where both LLS and BPCA outperform LSA, even though the entropy values of these data sets are relatively high. Possibly this is related to the smaller number of samples in these data sets; since there is less information in each individual gene for imputation, LSA, OLS, and KNN are not as effective as BPCA and LLS, which pull information from multiple genes simultaneously.

The entropy measure can be formally used to select an appropriate imputation algorithm for a particular data set, via the EBS scheme (6). It has an appealing advantage of fast computation since, for each new data set, the preferred method is selected only according to its entropy measure. The EBS scheme does have a practical limitation, in that it requires imputing MVs from multiple other data sets in order to fit the regression model (5). In addition its performance is dependent on which data sets were used to fit the model, although the cross validation results using the leave-one-out data sets demonstrate that the method performs well despite these potential limitations. One use of the EBS scheme is to reduce the number of imputation methods under consideration. For example, with low complexity data (*e*(*D*) < 0.9), we may restrict our attention to imputation methods like LSA, LLS, and BPCA, while with high complexity data (*e*(*D*) > 0.94) we may instead shift our attention to local imputation methods like LSA, KNN.c, and OLS..

The subset of methods selected by the EBS scheme can then be further evaluated by using the STS scheme to select the optimal imputation algorithm from among this reduced number. The STS scheme learns the structure of the expression data and selects the optimal imputation algorithm by self-training. This is accomplished by generating a small percentage of MVs among the genes with complete expression profiles to simulate the missing pattern in the original data, under the assumption that expression values are missing at random. Results from Simulation III indicate that this scheme picks the optimal or near-optimal imputation algorithm in every case. The selection scheme is sensitive to variation in the order of imputation ranking associated with variation in percentage of MVs, as evidenced by the selection of BPCA over LLS in SP.ELU and SP.AFA. However, in these cases the difference between the STS and optimal method in terms of imputation success was slight. In our experiments, we used 10 simulations to determine the best imputation algorithm in the STS scheme. This proved to be sufficient for distinguishing between the methods, as p-values from the rank-sum statistics were all on the order of magnitude of 10^-10 ^to 10^-12 ^In practice, even fewer simulations may be used to determine the best method, for example by using a sequential selection scheme where the set of imputation methods is evaluated after each iteration and "pruned" down until the best method is determined. This is a potential avenue of research that we will explore in the future. As noted above, the computational cost of STS can be further reduced by using the entropy of the data as a screening tool for the imputation algorithms, and when coupled in this fashion the two selection methods provide effective, complementary tools for determining the best imputation algorithm for a particular data set.

## Methods

### Data sets

We chose data from three fundamentally different experimental designs for our analyses, time series (TS), multiple exposure (ME), and time series × multiple exposure (TS × ME). Each of these experimental designs, in general, exhibit different types of expression patterns. As examples of TS experiments, we selected the yeast cell-cycle data from Spellman *et al.*[18] (both the alpha factor arrest [AFA] and elutriation [ELU] data sets) and the data reported in Baldwin *et al.*[19]. We selected the data sets from Alizadeh *et al.*[20], Alon *et al.*[21], Golub *et al.*[22], and Ross *et al.*[23] as examples of ME experiments. For TS × ME experiments, we selected the data sets from Gasch *et al.*[24], Hughes *et al.*[25], and Causton *et al.*[26]. Prior to analyses, all genes with missing and negative expression values were removed to create complete expression matrices, which were then natural log-transformed to facilitate scale-invariant comparisons across data sets. We denote by *D*_*j*_, *j *= 1,...,9 the expression matrices of the nine data sets: ALI, ALO, BAL, CAU, GAS, GOL, HUG, ROS, SP.AFA, and SP.ELU. A brief description of these data sets is given in Table 5.

**Table 5:** Descriptions of the nine microarray data sets used in our analysis.

Data set	Full Dim.	Used Dim.	Category	Organism	Expression Profiles
Alizadeh (ALI)	13412 × 40	5635 × 40	multiple exposure	H. sapiens	diffuse large B-cell lymphoma
Alon (ALO)	2000 × 62	2000 × 62	multiple exposure	H. sapiens	colon cancer and normal colon tissue
Baldwin (BAL)	16814 × 39	6838 × 39	time series, non-cyclic	H. sapiens	epithelial cellular response to L. monocytogenes
Causton (CAU)	4682 × 45	4616 × 45	multiple exposure × time series	S. cerevisiae	response to changes in extracellular environment
Gasch (GAS)	6152 × 174	2986 × 155	multiple exposure × time series	S. cerevisiae	cellular response to DNA-damaging adgents
Golub (GOL)	7129 × 72	1994 × 72	multiple exposure	H. sapiens	acute lymphoblastic leukemia
Ross (ROS)	9706 × 60	2266 × 60	multiple exposure	H. sapiens	NCI60 cancer cell lines
Spellman, AFA (SP.AFA)	7681 × 18	4480 × 18	time series, cyclic	S. cerevisiae	cell-cycle genes
Spellman, ELU (SP.ELU)	7681 × 14	5766 × 14	time series, cyclic	S. cerevisiae	cell-cycle genes

### Imputation algorithms

#### K-Nearest Neighbors (KNN)

The widely used KNN procedure finds the *k *genes that are most similar to the gene with the MV as determined by a distance metric, most frequently Euclidean distance or Pearson correlation. The MV is then estimated as the average of these *k *neighbor genes for the same array, weighted according to the inverse of their distance [1]. Because neighbors determined by a correlation-based distance may be highly correlated, but different in magnitude, we first standardize the genes to mean zero, standard deviation one prior to neighbor-selection, and then re-scale them to the original scale following imputation, to account for this situation. The distance used to select the neighbors is 1 - *r*, where *r *is the Pearson correlation. The normalization/re-scaling process is unnecessary for Euclidean-based neighbor selection, because neighbors with similar magnitude to the gene with the MV are used for imputation. For both neighbor selection methods, MVs are omitted from the distance calculation, so that it is based only on the complete pairwise observations between two genes. We tested both Euclidean (KNN.e) and correlation (KNN.c) based neighbor selection approaches in our study..

#### Ordinary Least Squares (OLS)

In this neighboring-based approach, the gene with the MV is regressed over each of the *k *most similar neighbor genes. MVs are imputed as the weighted average of the predicted values from the regression of the gene with MVs onto each neighbor gene [8,9]. Neighbors are selected based on the absolute Pearson correlation, and the weight we use is the same as in [9],

$$w={\left(\frac{r_{yx}^{2}}{1-r_{yx}^{2}+10^{-6}}\right)}^{2},$$

where *r*_*yx *_is the correlation between *y*, the gene with MVs, and the potential neighbor gene *x*. As in KNN, MVs are omitted from the distance calculation and the simple linear regressions.

#### Local Least Square (LLS)

The LLS procedure of [10] selects neighbors based on the Pearson correlation as in OLS, but instead of weighting univariate regressions they perform multiple regression using all *k *nearest neighbors. The MVs are imputed based on the least squares estimates, determined using the pseudoinverse of the *k *nearest neighbors expression matrix. If the percentage of MVs is relatively small, then neighbor genes with MVs are excluded from the least squares system, otherwise MVs are initially estimated by the row (gene-wise) average.

#### Least Squares Adaptive (LSA)

The LSA procedure of [9] combines gene-based and array-based imputation estimates, using an adaptive procedure to determine the weighting of the two estimates. The gene-based estimates are determined as in OLS, and the array-based estimates are determined by multiple regression based on the arrays, where the gene-based estimates are substituted for the MVs in the expression matrix. To determine the best weighting of the two estimates, known values in the data matrix are initially re-estimated, and the errors of the gene- and array-based estimates are determined. The optimal weight is determined by minimizing the sum of the squared errors for the re-estimated data. The weights are determined adaptively by considering the strength of the gene correlation in the gene-based estimates. That is, only genes with similar values of the maximum gene absolute correlation used in the gene-based estimation are factored into the weight calculation.

#### Partial Least Squares (PLS)

PLS regression selects linear combinations of genes (called components) exhibiting high covariance with the gene having the MV (the target gene). The first linear combination has the highest covariance with the target gene, and subsequent components have the greatest covariance with the target gene in a direction orthogonal to the previously selected components until a total number of *c *components are selected. The missing values are then imputed by regressing the target gene onto the PLS components. MVs are first imputed by row average prior to PLS imputation [8].

#### Singular Value Decoposition (SVD)

This approach initially sets MVs to the row average, and then uses singular value decomposition of the gene expression matrix to create orthogonal principle components, called "eigengenes." The proportion *p *of eigengenes which correspond to the largest eigenvalues are then used to reconstruct the MVs in the expression matrix. An expectation-maximization (EM) approach is used to iteratively improve the imputed MVs and expression covariance matrix until total change in the matrix falls below a prescribed threshold (here taken to be 0.01) [1].

#### Bayesian Principal Component Analysis (BPCA)

This method uses Bayesian estimation to fit a probabilistic PCA model [2]. A variational Bayes algorithm is used to iteratively estimate the posterior distribution of the model parameters and the MVs until convergence is reached. The key feature of this approach is that principal axes with small signal to noise ratios are shrunk toward zero, so that the algorithm automatically screens for those axes that are the most relevant. MVs are initially imputed by row or gene-wise average.

### Assessment of Performance

We employed the log-transformed root mean squared error (LRMSE) as the metric by which we assessed the performance of our imputation methods. This statistic has the important property of scale-invariance, which allows direct comparisons of imputation accuracy across different data sets. Therefore, it is scientifically more reasonable than other RMSE metrics. By log-transforming the expression matrices prior to imputation *y*_*ij *_= log(*x*_*ij*_), where *x*_*ij *_is the expression intensity of gene *i *and sample *j*), we calculate the LRMSE as the ordinary RMSE of the log-transformed expression matrix (where ${\hat{y}}_{ij}$ is the imputed value of *y*_*ij*_):

(1) $$LRMSE=\sqrt{\frac{\sum_{\{i,j:x_{ij}\text{ missing\}}}{\left({\hat{y}}_{ij}-y_{ij}\right)}^{2}}{\#\{x_{ij}\text{ missing}\}}}.$$

### Evaluation for selection schemes

Before introducing our two selection schemes for determining the preferred imputation method, we demonstrate two measures for evaluating the performance of a selection scheme. Given a complete expression matrix *D*, we create *N *data sets with 5% random missing values, denoted by *D*^(*k*)^, *k *= 1,..., *N*. The 5% missing values in *D*^(*k*) ^are imputed by method *M*, and the resulting imputed data are denoted by ${\hat{D}}_{M}^{\left(k\right)}$. The LRMSE of the imputed data ${\hat{D}}_{M}^{\left(k\right)}$ compared to the original data *D *is denoted by $LRMSE\left({\hat{D}}_{M}^{\left(k\right)},D\right)$. We define

$$M^{\left(k\right)}=\arg\min_{M}LRMSE\left({\hat{D}}_{M}^{\left(k\right)},D\right)$$

to be the optimal imputation method for data *D*^(*k*) ^judging from the original complete data *D*, and treat it as the gold standard optimal method. Given any selection scheme *S*, we can use the following two indexes to measure the degree of deviation of the methods selected by *S *and the optimal methods *M*^(*k*)^:

(2) $$LRMSE_{Diff}(S,D)=\frac{1}{N}\sum_{k=1}^{N}\left[LRMSE\left({\hat{D}}_{S(D^{\left(k\right)})}^{\left(k\right)},D\right)-LRMSE\left({\hat{D}}_{M^{\left(k\right)}}^{\left(k\right)},D\right)\right]$$

(3) $$Accuracy(S,D)=\frac{1}{N}\sum_{k=1}^{N}{\text{I}}_{\left\{S\left(D^{\left(k\right)}\right)=M^{\left(k\right)}\right\}},$$

where the indicator function ${\text{I}}_{\left\{S\left(D^{\left(k\right)}\right)=M^{\left(k\right)}\right\}}$ takes the value one if the selected method for data *D*^(*k*) ^matches the true optimal method *M*^(*k*) ^and zero otherwise. The first measure calculates the average difference in LRMSE values between the selected method and the optimal method, and the second measure determines how frequently the selected method coincides with the optimal method.

### Entropy-based selection (EBS)

In our analysis of the nine microarray data sets, we observed that the performance of different imputation methods on a data set is related to the data complexity, which can be summarized by the entropy of the eigenvalues of the covariance matrix in the data. To be more explicit, for an expression matrix *D*, we first calculate the eigenvalues of the covariance matrix, *λ*_*i*_, *i *= 1,..., *k*, where *k *is the rank of the covariance matrix. The complexity of the data set *D *is calculated as the following entropy measure

(4) $$e(D)=-\frac{\sum_{i=1}^{k}p_{i}\log p_{i}}{\log(k)},$$

where $p_{i}=\sqrt{\lambda_{i}}/\sum_{l=1}^{k}\sqrt{\lambda_{l}}$.

Note that the term log(*k*) in the denominator is to standardize for different *k *and represents the summand in the case where *p*_*i *_= 1/*k*, 1 ≤ *i *≤ *k*. It is easy to show that 0 ≤ *e*(*D*) ≤ 1. Intuitively, a low entropy measure reflects low complexity of the data, represented by relatively few eigenvalues that are distinctively larger than the other eigenvalues. This indicates that the data matrix can be effectively reduced to a low-dimensional space. Conversely, a large entropy measure indicates a similar magnitude of all the eigenvalues and that the data cannot be reduced to a low-dimensional space. In the case of chaotic data, all the eigenvalues are the same and (4) gives a maximum entropy of one regardless of the rank.

We relate the entropy level of a data set to the performance of each imputation method by fitting a linear model of the form

(5) $$LRMSE\left({\hat{D}}_{j;M_{i}}^{\left(k\right)},D_{j}\right)=\alpha_{0}+\alpha_{i}+\beta_{i}e(D_{j})+\gamma_{j}+\varepsilon_{ijk},$$

where $\sum_{i=1}^{8}\alpha_{i}=0$, $\sum_{i=1}^{8}\beta_{i}=0$, $\sum_{j=1}^{10}\gamma_{j}=0$, $LRMSE\left({\hat{D}}_{j;M_{i}}^{\left(k\right)},D_{j}\right)$ is the LRMSE for the data sets with intentional missing values $D_{j}^{\left(k\right)}$ imputed by method *M*_*i*_, *e*(*D*_*j*_) is the entropy level of *D*_*j*_, *α*_*i *_and *β*_*i *_are the intercept and regression slope for imputation method *M*_*i*_, *i *= 1, ...,8 *y*_*j *_is a fixed effect representing the intrinsic imputation difficulty of data set *D*_*j*_, and *ε*_*ijk *_are random noises. Essentially, the above model is an analysis of covariance model, with separate regression lines fitted for each method of the LRMSE values on the entropy values of the data sets. Note that the parameter *y*_*j *_in model (5) is necessary because different data sets *D*_*j *_have different levels of intrinsic imputation difficulty. We fit the model in equation (5) using all nine data sets and denote the estimated model by *L*(*D*_1_,...,*D*_9_), with ${\hat{\alpha }}_{i}$ and ${\hat{\beta }}_{i}$ the resulting estimates. Intuitively if ${\hat{\beta }}_{i}$, >> 0 the imputation method *M*_*i *_performs better (with smaller LRMSE) for data sets with low entropy measure. Conversely, if ${\hat{\beta }}_{i}$ << 0, *M*_*i *_is better for more complex (with higher entropy) data sets.

Given a new data set *D *with missing values, we can determine the best imputation method in *M*_*i *_from the above estimated linear model. We denote by ${\hat{D}}_{M_{i}}$ the imputed complete data set from data set *D *with missing values imputed by *M*_*i*_. Since the entropy of *D*, *e*(*D*), cannot be calculated with missing values, it is estimated by $\tilde{e}(D)=e({\hat{D}}_{M_{Ave}})$, where *M*_*Ave *_is the imputation method based on the average expression level of a gene. From (5), the best imputation method for data set *D *is selected by

$${\hat{M}}_{EBS}\left(D,L(D_{1},...,D_{10})\right)=\arg\min_{M_{i}}{\hat{\alpha }}_{i}+{\hat{\beta }}_{i}\tilde{e}(D),$$

i.e., the imputation method with the lowest predicted LRMSE of data *D *based on the model fitted from the nine selected data sets, *L*(*D*_1_,..., *D*_9_).

We performed cross validation to evaluate the above entropy-based selection scheme. For each *D*_*j*_, let *D*_(*j*) _= {*D*_*i*_, *i *= 1,...,9}\*D*_*j *_denote the "leave-one-out" data sets consisting of the simulations from all other data sets except *D*_*j *_. We fit the linear model (5) using the set *D*_(*j*) _and define the EBS scheme for each data set with intentional missing values $D_{j}^{\left(k\right)}$ as:

(6) $$S_{EBS}(D_{j}^{\left(k\right)})={\hat{M}}_{EBS}\left(D_{j}^{\left(k\right)},L(D_{\left(j\right)})\right).$$

The EBS scheme can then be evaluated by the difference (2) and accuracy (3) measures defined previously.

### Self-training selection (STS)

The STS procedure explicitly determines the optimal imputation algorithm for a particular data set by simulating MVs in the subset of the expression matrix which is complete (i.e., contains no MVs), imputing these simulated MVs, and comparing these imputed values to the known expression values. This strategy has also been employed by others, though for different purposes. Jornsten *et al.*[11] used the idea to find a convex combination of the imputation methods, while Kim *et al.*[10] found the optimal number of nearest neighbors for LLS imputation. The rank of each imputation method, in terms of LRMSE, is noted in each simulation and the method with the smallest rank-sum statistic over multiple simulated data sets is selected.

More specifically, we randomly remove an additional 5% of expression values from each $D_{j}^{\left(k\right)}$, and perform *n *replicates to generate data sets $D_{j}^{\left(k)(l\right)}$, *l *= 1,..., *n*. For each method *M*_*i*_, we calculate the rank-sum statistic

$$R(M_{i},D_{j}^{\left(k\right)})=\int_{l=1}^{n}Rank_{M_{i}}\left(LRMSE\left({\hat{D}}_{j;M_{i}}^{\left(k)(l\right)},D_{j}^{\left(k\right)}\right)\right).$$

The STS scheme is then defined as

(7) $$S_{STS}(D_{j}^{\left(k\right)})=\arg\min_{M_{i}}R(M_{i},D_{j}^{\left(k\right)}).$$

Again, the difference (2) and accuracy (3) measures defined previously can be applied for evaluation. To assess whether the *n *= 10 replicates we used was sufficient for determining the preferred imputation method, the null hypothesis that all methods are equally effective (i.e., the rank-sum statistics are all identical) was tested using Friedman's test [27].

### Simulations for evaluation

To evaluate our imputation methods and selection schemes on missing data, we randomly removed known expression values from the complete matrices, imputed these intentionally created MVs, and assessed imputation performance using the LRMSE. For our first set of simulations (Simulation I), we empirically tested 10 simulations for each data set under different parameter values for each of the methods to optimize their performance, with the percentage of MVs set at 5%. For the KNN and OLS methods, we tested values of *k *between 5 and 200, while for SVD we varied the proportion *p *of eigengenes between 0.1 and 0.5. For PLS we tested the number of components *c *between 2 and 25. LLS has parameter optimization built into the algorithm. The number of neighbor genes evaluated depended on the data set, but generally varied from 10 to upwards of 2000, with spacing between adjacent *k *values increasing as *k *increased. For our study, we ran the algorithm several times to determine the optimal *k *value, then held this fixed for the subsequent simulations. BPCA and LSA do not require parameter optimization.

We next performed 50 simulations using the optimized parameters on different data sets with 5% of the expression values removed to compare the accuracy of different algorithms across a wide range of microarray data (Simulation II). These 50 simulations were also used to test the EBS scheme via the leave-one-out data sets cross validation. To test the STS scheme, we ran an additional set of simulations (Simulation III). Simulation III consisted of 10 initial "first tier" simulations per data set each having 5% MVs. For each of these first tier simulations, 10 "second tier" simulations having an additional 5% MVs each were generated. The second tier simulations were used to determine the STS selected algorithm, while the first tier simulations were used to evaluate the performance of STS. Lastly, we ran an additional 20 simulations at 2%, 10%, and 15% missing for the ALO, CAU, GOL, SP.AFA, and SP.ELU data sets, to evaluate the performance of each algorithm with varying percentage of MVs.

## Availability and Requirements

KNN, OLS, PLS, SVD, and BPCA were all coded in the R language [28]. The BPCA code was ported from the original Matlab code provided at [29]. LLS is available at [30], and LSA at [31].

## Competing interests

The author(s) declares that there are no competing interests.

## Authors' contributions

GNB wrote the code for the imputation algorithms and the selection schemes, analyzed the results, conducted the simulation studies, and drafted the manuscript. JRS also conducted the simulation studies, tabulated the results, and drafted the manuscript. REB helped code the imputation algorithms. MJL performed the data collection. GCT conceived of the study and the selection schemes, participated in its design and coordination, and helped to draft the manuscript. All authors read and approved the final manuscript.
